# First detection of *Rumenfilaria andersoni* in semi-domesticated reindeer (*Rangifer tarandus tarandus*) in Norway

**DOI:** 10.1186/s13028-026-00893-2

**Published:** 2026-11-10

**Authors:** Jason Byrne, Kayla J. Buhler, Ingebjørg H. Nymo, Monica A. Sundset, Stine Bull-Aurbakken, Ester Malmström, Henna Kaarakainen, Sauli Laaksonen, Morten Tryland, Rebecca K. Davidson

**Affiliations:** 1 Department of Arctic and Marine Biology, UiT — The Arctic University of Norway, Tromsø, 9037 Norway; 2 Department of Forestry and Wildlife Management, University of Inland Norway, Koppang, 2480 Norway; 3 Section for Terrestrial Animal and Wildlife Health, Department of Animal Health, Norwegian Veterinary Institute, Tromsø, 9016 Norway; 4 Department of Veterinary Services, Municipality of Inari, Latutie 1, Ivalo, 99800 Finland; 5 Department of Veterinary Biosciences, Faculty of Veterinary Medicine, University of Helsinki, Helsinki, 00014 Finland

**Keywords:** Climate-sensitive infection, Filarioid nematodes, Modified Knott’s assay, Microfilaria, *Setaria tundra*

## Abstract

Filarioid nematodes, including *Rumenfilaria andersoni* and *Setaria tundra*, are vector-borne parasites of cervids in Fennoscandia. We screened blood samples collected December 2024 – February 2025 from three herds of semi-domesticated reindeer (*Rangifer tarandus tarandus*) in middle and northern Norway (Trøndelag; *n* = 27, Nordland; *n* = 16; Finnmark; *n* = 28). Samples were examined for microfilariae using a Modified Knott’s Assay, followed by PCR and Sanger sequencing of a partial mitochondrial *COX1* gene for species confirmation. Microfilariae morphologically consistent with *R. andersoni* were detected in three animals from Finnmark (*n* = 3/28, 10.7%, [95% confidence interval (CI) 3.7–27.2]) with a mean intensity of 353 microfilaria per mL (mf/mL, range 63–881). There was no evidence of *S. tundra* microfilariae in the same animals (*n* = 0/28, 0% [95% CI 0.0–12.1]). No microfilariae were detected in the animals from Trøndelag (*n* = 0/27, 0% [95% CI 0.0–12.5]) or Nordland (*n* = 0/16, 0% [95% CI 0.0–19.4]). *COX1* sequencing confirmed *R. andersoni* and showed 100% identity with a reindeer-derived isolate previously reported from Finland. These findings provide the first confirmed record of *R. andersoni* in Norway and expand the known distribution of this climate-sensitive parasite, underscoring the need for targeted surveillance to clarify its occurrence, transmission dynamics, and potential health and management implications for semi-domesticated reindeer.

## Findings

*Rumenfilaria andersoni* and *Setaria tundra* are filarioid nematode parasites reported in Fennoscandian cervids [1, 2]. *Rumenfilaria andersoni* was first documented in Finland and was likely introduced from North America through the importation of white-tailed deer (*Odocoileus virginianus*) in 1935 [2, 3]. It has since been detected in moose (*Alces alces*), white-tailed deer, roe deer (*Capreolus capreolus*), and reindeer (*Rangifer tarandus tarandus*) in Finland, and moose in Sweden [2]. *Setaria tundra* was first described in reindeer in Russia in 1928 [4]. It has been associated with reindeer disease outbreaks across Fennoscandia in the 1970s, with further epidemics in Finland in the early 2000s and onwards [5]. In Finland, *S. tundra* has also been detected in wild forest reindeer (*Rangifer t. fennicus*), roe deer, and moose [6].

Transmission of these parasites rely on a biting insect-cervid life cycle. Microfilariae ingested by the insect vector during a blood meal, develop to infective L3 larvae, and are transmitted back to cervids during subsequent blood meals [2, 7, 8]. In cervids, *R. andersoni* adults localise in ruminal lymph vessels [2], while *S. tundra* adults establish in the abdominal cavity [1]. Both species release blood-circulating microfilariae, sustaining the transmission cycle [8].

Mature *R. andersoni* infection can cause localised inflammation around the ruminal lymph vessels, but overall pathogenic consequences are obscure [2]. The intermediate host for *R. andersoni* remains unknown, although nocturnal periodicity of microfilaremia suggests a night-biting insect [2]. Unlike *S. tundra*, ivermectin is ineffective against adult stages of *R. andersoni*, but demonstrates elimination of microfilaremia for approximately two months in adult reindeer treated in November [2, 9].

This study investigated the occurrence of microfilariae in blood samples from reindeer in the middle and north of Norway. Three-year-old female reindeer from herds in Finnmark (*n* = 28), Nordland (*n* = 16) and Trøndelag (*n* = 27) counties were sampled between December 2024 and February 2025. All sampled reindeer were considered clinically healthy at the time of sampling and all animal procedures were performed in accordance with the Norwegian Animal Welfare Act and approved by the National Animal Research Authority (FOTS ID: 29948). Blood samples were obtained from the jugular vein as previously described [10] in BD Vacutainer^®^ K2E 10 ml EDTA tubes and stored at -20 °C pending analysis.

The Modified Knott’s Assay was performed following the American Heartworm Society Guidelines [11], with slight modifications. Formalin was substituted with distilled water, and thorough mixing steps using 10x sample inversions and vortexing (2500 rpm, 3 × 3 s) were utilised to optimise sample homogeneity [12]. The stained microfilariae were visualised at 100x magnification for counting and 400x magnification for morphometric measurement and identification [11]. As blood samples were frozen prior to analysis, identification was interpreted cautiously and supported by morphometric measurements. The microfilaria count per sample was calculated and reported as microfilariae per millilitre of blood (mf/mL). The remaining Modified Knott’s Assay pellets were stored at -20 °C pending molecular analysis. Prevalence confidence intervals were calculated using the Wilson confidence interval calculator [13].

DNA extraction was performed on the Modified Knott’s Assay pellets using the DNeasy Blood & Tissue Extraction Kit (Qiagen, Hilden, Germany) following the Spin-column protocol (Purification of Total DNA Tissue from Animal Tissues), with adjustments to Proteinase K incubation time (56 °C, 16 h) and DNA elution buffer volume (30 µL) [14]. The extraction controls consisted of a ≈ 1 mm segment of a mature *S. tundra* (positive control) and PCR-grade water (negative control). The DNA concentration and quality were verified using a NanoDrop^®^ 2000c UV Visible Spectrophotometer (Thermo Fisher Scientific, Waltham MA, USA).

PCR amplification of a partial cytochrome oxidase c subunit 1 (COX1) gene was performed using primers COIintF (5′-TGA TTG GTG GTT TTG GTA A-3′) and COIintR (5′-ATA AGT ACG AGT ATC AAT ATC-3′) [15, 16]. PCR was run in duplicate using 2X Taq Plus Master Mix (VWR, Radnor, PA, USA). Each run included a no template control, a negative extraction control, and a positive control. Products were visualised on 2% ethidium bromide-stained agarose gel (ChemiDoc™ Go Imaging System, Bio-Rad, Hercules CA, USA). PCR products were excised using the E.Z.N.A^®^ Gel Extraction Kit (Omega Bio-Tek, Norcross GA, USA) following manufacturer’s instructions [17]. Due to non-specific amplification of off-target sites, and loss of DNA in the purification process, products from multiple wells were pooled, re-purified and concentrated using the QIAQuick PCR Purification Kit (Qiagen), with final elution in 30 µL Buffer EB [18].

Sanger sequencing was prepared using the BigDye™ Terminator v3.1 Cycle Sequencing Kit (Thermo Fisher Scientific Baltics UAB, Vilnius, Lithuania) following manufacturer’s instructions and sequenced (Sequence Lab, University Hospital of Northern Norway, Tromsø, Norway) [19]. The forward and reverse sequences were aligned using Chromas (Technelysium Pty Ltd, South Brisbane, Australia) and MEGA12, and regions of sequence similarity were identified via the Basic Local Alignment Search Tool (BLAST) in the GenBank database [20, 21].

**Fig. 1:**
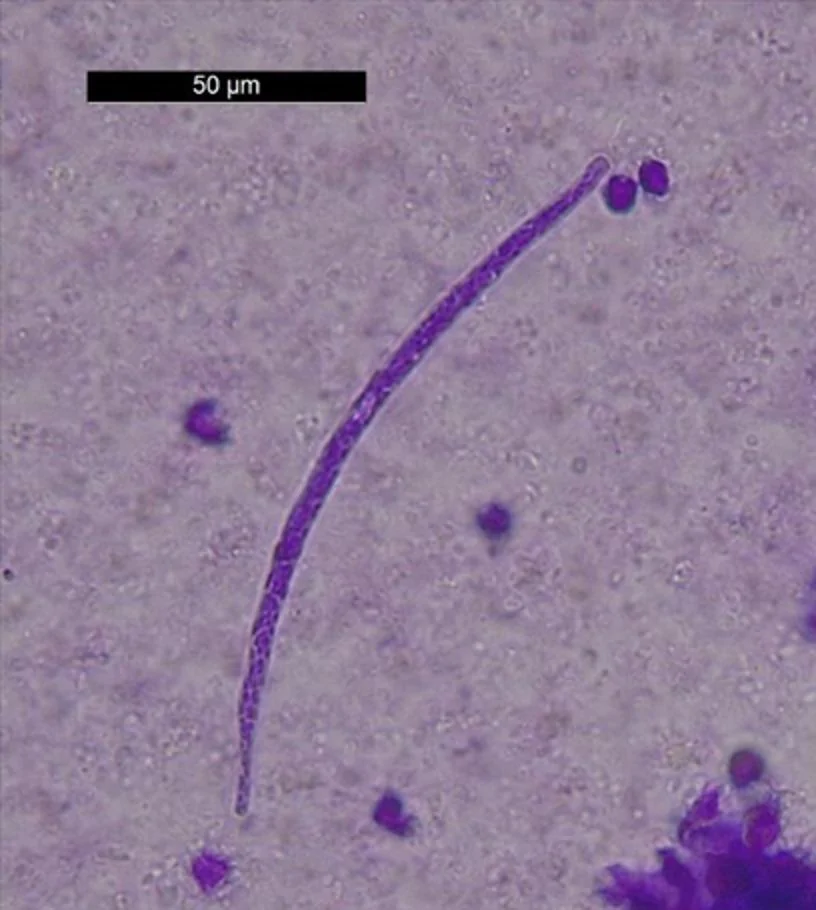
“*Rumenfilaria andersoni* microfilaria”; *Rumenfilaria andersoni* microfilaria detected in a semi-domesticated reindeer (*Rangifer tarandus tarandus*) blood sample from Finnmark through the Modified Knott’s Assay at 400x magnification. Microfilaria length ≈ 150 μm. Size bar = 50 μm. Photo: Jason Byrne

*Rumenfilaria andersoni* was detected with the Modified Knott’s Assay in blood samples from the Finnmark animals (*n* = 3/28, 10.7% [95% CI 3.7–27.2]) (Fig. 1). The overall mean intensity in the three positives was 353 mf/ml (range 63–881). *Rumenfilaria andersoni* was not detected in reindeer from Nordland (*n* = 0/16, 0% [95% CI 0.0–19.4]) or Trøndelag (*n* = 0/27, 0% [95% CI 0.0–12.5]). *Setaria tundra* was not detected in any blood sample using the Modified Knott’s Assay (*n* = 0/71, 0% [95% CI 0.0–5.1]).

Although off-target amplification occurred, high-quality bidirectional sequence data were obtained, yielding a 564 bp *COX1* sequence (GenBank accession no. PX501989) which showed 100% identity with an *R. andersoni COX1* sequence previously obtained from a Finnish reindeer (GenBank accession no. JQ888273.1).

This first detection of *R. andersoni* in Norway demonstrates that the parasite is present in at least one semi-domesticated reindeer herd in Finnmark County. The lack of previous records does not preclude earlier presence, especially considering that infections causing limited pathology are less likely to be detected in the absence of targeted surveillance.

The detection in Finnmark may reflect range expansion from Sweden and Finland where the prevalence and distribution of filarioid parasites has been increasing [2, 22] This range expansion has likely been facilitated by climate-driven changes in vector ecology. Warmer summers impact biting insect activity and spatiotemporal population dynamics, potentially enabling transmission in locations previously unaffected by *R. andersoni* [23]. Therefore, the appearance of *R. andersoni* in Finnmark may be a potential warning signal for climate-associated shifts in vector-borne parasite ranges in northern Fennoscandia.

Comparison of prevalence and abundance data must account for the sampling month given the long prepatent period of both parasites. Sampling of the *R. andersoni*-positive Finnmark herd occurred in December. It is possible to detect initial infections in calves as early as November, whereas adult reindeer infections may be detected in autumn due to potential infection persistence from the previous summer infection window [2, 22].

The prevalence of *R. andersoni* microfilaremia in adult reindeer in Finnmark in December 2024 was relatively low (10.7%) compared with the prevalence reported in adult reindeer in Finland during winter sampling in 2003–2004 and 2005–2006 (76%) [2]. This difference in prevalence, and a slightly lower infection intensity (Finnmark: mean 353 mf/ml, range 63–881; Finland: mean 678 mf/ml, range 1–19400) is difficult to interpret directly, but could reflect recent range expansion and establishment. However, local environmental constraints on transmission, differences in vector communities, or differences in seasonal exposure times, as well as demographic (age, sex) and temporal differences in the populations sampled rather than a true difference in parasite performance could also explain some of the differences seen. Additional sampling in Finnmark later in winter may have revealed an increased prevalence, given the long prepatent period (≈ 5 months) of *R. andersoni* [2].

Ivermectin treatment was routinely carried out in this herd each winter, potentially impacting *R. andersoni* microfilaria abundance in blood samples analysed. As *S. tundra* is sensitive to ivermectin, the lack of detection of this parasite in this herd may indicate effective treatment rather than a true absence. *Setaria tundra* has been documented in northern reindeer herds in Finland and Sweden [5, 22], and as of late 2025, both parasites were detected at slaughter from herds across Finnmark [24]. Further investigations are needed to ascertain the geographic distribution, abundance and prevalence of these parasites in northern Norway.

The limitations of this work include small sample sizes in adult females only, single-year analysis, and possibly seasonal effects on microfilaremia. In addition, only one *COX1* sequence was obtained, precluding assessment of genetic diversity and phylogenetic relationships among *R. andersoni* isolates. Broader, multi-month and multi-year surveillance and vector studies, as well as more systematic *post-mortem* registration of findings during commercial slaughter, are needed to clarify transmission patterns and the implications for reindeer health. Further work is also needed to assess how the infection dynamics reflect climate-sensitive parasites and vector responses to changing environmental conditions. Given the off-target amplification that was seen, future studies may consider re-evaluating primer design especially when working with *Rangifer*.

Ultimately, *R. andersoni* was detected in a semi-domesticated reindeer herd in Finnmark, representing the first confirmed record in Norway. No microfilariae were detected in the two herds further south and no evidence of *S. tundra* was found. The findings in this study and more recent records of both *R. andersoni* and *S. tundra* in Finnmark/Norway highlights the need for expanded surveillance of these climate-sensitive parasites to clarify the distribution and drivers of transmission, as well as their potential impact on reindeer health and management.

## Data Availability

The datasets used and/or analysed during the current study are available from the corresponding author on reasonable request.

## Declarations

### Prior publication

Data included in this article have previously been presented on the Nordic Reindeer Husbandry Conference, in Alta, Norway 12–14 February 2025.
